# Assessing the Effectiveness of Frequency Manoeuvring in UAV Networks Under Jamming and Interference

**DOI:** 10.3390/s26154785

**Published:** 2026-07-28

**Authors:** Piotr Targowski, Sebastian Łeska, Jakub Walczak, Szymon Chmielewski, Janusz Furtak

**Affiliations:** Faculty of Cybernetics, Military University of Technology, 00-908 Warsaw, Poland; sebastian.leska@wat.edu.pl (S.Ł.); jakub.walczak01@wat.edu.pl (J.W.); szymon.chmielewski01@wat.edu.pl (S.C.); janusz.furtak@wat.edu.pl (J.F.)

**Keywords:** UAV networks, frequency manoeuvring, anti-jamming, EMANE, CORE, packet delivery ratio, frequency hopping, adaptive bandwidth, contested electromagnetic environment

## Abstract

**Highlights:**

**What are the main findings?**

- Frequency manoeuvring is beneficial only under degraded radio conditions.
- Immediate switching performs best under environmental interference, while periodic hopping achieves the highest packet delivery ratio under jamming and combined interference: a 12.2-percentage-point PDR gain over a fixed channel under jamming and a 6.7-point gain under combined interference.

**What are the implications of the main findings?**

- The manoeuvring strategy for UAV networks should be matched to the interference type and mission phase.
- Packet delivery ratio alone is insufficient for policy selection, because coverage continuity, latency and jitter can lead to different operational choices.

**Abstract:**

This paper investigates frequency manoeuvring as a method to improve the resilience of unmanned aerial vehicle (UAV) networks operating in contested electromagnetic environments. The study considers scenarios in which the network initially operates on a single channel and is then exposed to intentional jamming or unintentional interference affecting the primary channel, adjacent channels or a wider frequency range. Several response policies are compared, including no channel change, immediate switching after quality degradation is detected, delayed switching after a defined loss-of-connectivity interval, and periodic frequency hopping. In addition to channel switching, the analysis also considers changes in channel bandwidth, comparing narrower channels with lower throughput but potentially higher resistance to interference against wider channels with greater capacity but increased susceptibility to disruption. The evaluation includes the switching cost, which is modelled as temporary packet loss, additional delay and jitter during reconfiguration. Performance is assessed using the packet delivery ratio, latency, jitter, packet loss and communication continuity. The main objective is to identify the interference conditions under which frequency manoeuvring becomes operationally beneficial and to determine which policy offers the best trade-off between resilience and communication performance. In quantitative terms, immediate switching under environmental interference achieved a PDR of 0.961 and a mean latency of 123.6 ms compared with a PDR of 0.946 and a mean latency of 138.7 ms for fixed-channel operation. Manoeuvring gave a substantial 12.2-percentage-point PDR gain under jamming (periodic hopping: 0.780 vs. 0.658) and a 6.7-percentage-point gain under combined interference (0.674 vs. 0.607). These results indicate that manoeuvring is most worthwhile once interference is persistent and channel-focused rather than purely environmental.

## 1. Introduction

Unmanned aerial vehicles (UAVs) are now routinely employed across military reconnaissance, border and infrastructure monitoring, disaster response and civil inspection missions, and this growth has made robust wireless connectivity a first-order design concern rather than an afterthought. As UAV teams increasingly operate as networked systems instead of single remotely piloted platforms, the electromagnetic environment in which they communicate has become an operational variable in its own right that is shaped by both unintentional interference and deliberate denial-of-service action.

UAV reconnaissance, surveillance and tactical support missions increasingly rely on wireless networking rather than isolated point-to-point control links. Multi-UAV teams, relays and mobile ground control stations can extend observation range, maintain situational awareness and support rapid re-tasking. Still, they also expose the mission to the quality of the shared electromagnetic environment. A radio network that performs well in a clean test range can become fragile when the same assets encounter a moving jammer, a wideband environmental disturbance, hidden-node effects, spectrum congestion or rapid geometry changes caused by mobility.

This problem is visible in two related research areas. First, UAV networking studies emphasise strong line-of-sight potential, high mobility and unstable topology [1,2,3,4,5]. Second, wireless security studies show that jamming is a practical denial-of-service mechanism because it can degrade reception, acknowledgements, and carrier sensing without compromising higher-layer cryptographic protection [6,7,8,9]. For reconnaissance and unmanned-vehicle missions, these concerns converge: the most valuable link is often a mobile, low-power, bandwidth-limited channel that carries telemetry, tasking and sensor updates.

Frequency manoeuvring is a natural countermeasure. A network may remain on a fixed channel until degradation is detected, switch immediately after a quality threshold is crossed, wait for repeated failures to avoid reacting to transient fades, hop periodically regardless of local observations, or adapt channel bandwidth to reduce overlap with a disturbance. These mechanisms are familiar from cognitive radio, dynamic spectrum access and spread-spectrum communication [10,11,12,13,14,15]. However, their operational value in a UAV mission depends on details that are often hidden in abstract models: the switching cost, the geometry of the disturbance, the number of communicating nodes, the message pattern, the temporal persistence of interference, and the mission’s continuity requirement.

This paper addresses the following question: under what interference conditions does frequency manoeuvring become more beneficial than staying on a fixed channel?

The contribution is threefold:

- An EMANE/CORE-based experimentation workflow for UAV communication scenarios with mobility, environmental interference, jamming profiles and automated repetitions;
- A comparative evaluation of five frequency-control policies across clean, environmental, jamming-only and combined-interference settings;
- An operational interpretation of the trade-off between PDR (Packet Delivery Ratio), latency, jitter and coverage continuity when switching itself introduces loss, delay and jitter.

The novelty relative to prior UAV anti-jamming and cognitive-radio work (Section 2) is not a new physical-layer algorithm but rather a controlled, repeatable measurement of when simple, interpretable manoeuvring rules are worth their reconfiguration cost under mission-representative traffic. Concretely, interference intensity is operationalised through four classes with explicitly reported parameter ranges (clean; environmental, with radius, packet-loss and delay contributions; jamming, with an intentional jammer characterised by radius, strength, packet-loss/ACK-loss contribution, frequency and bandwidth; and combined); task types correspond to command, tasking, telemetry and ISR-update messages with critical or high mission priority (Section 3.1); and the network-layer assumption is a fixed, single-hop-per-link application-messaging topology without a dynamic routing protocol so that the measured effect is attributable to the frequency/bandwidth policy rather than to routing convergence (Section 2.1).

The remainder of this paper is organised as follows. Section 2 reviews UAV networking, jamming and experimentation tools. Section 3 describes the scenario model, manoeuvring policies and metrics. Section 4 presents the results. Section 5 discusses operational implications and limitations. Section 6 concludes the paper.

## 2. Related Work

### 2.1. UAV and Flying Ad Hoc Networks

UAV communication networks differ from classical mobile ad hoc networks because mobility is fast, mostly three-dimensional and mission-driven. Early FANET surveys identified topology dynamics, intermittent links, energy constraints and routing stability as central problems [1]. Later surveys connected these problems to civil and tactical UAV applications, including surveillance, disaster response, infrastructure inspection and relay support [2,4]. Air–ground channel properties can be favourable due to line-of-sight propagation, yet this advantage does not eliminate vulnerability to interference, blockage, antenna orientation, or adversarial action [3,5].

The UAV communication literature also distinguishes between cellular UAV-to-X communication and ad hoc/mission-network operation [16,17]. Cellular integration offers managed spectrum, infrastructure support and standardised mobility control, whereas a tactical UAV team may need to operate with limited or no infrastructure, using relays and local coordination. In the latter case, network-layer protocols such as AODV (Ad hoc On-Demand Distance Vector), OLSR (Optimised Link State Routing), and DSR (Dynamic Source Routing) remain relevant baselines for dynamic routing [18,19,20]. At the same time, link-layer behaviour remains closely tied to IEEE 802.11-style contention and channel conditions [21,22]. The present work does not compare routing protocols; instead, it isolates a lower-layer operational question: whether changing frequency and bandwidth improves communication continuity when the radio environment deteriorates.

### 2.2. Jamming, Interference and Anti-Jamming

Jamming attacks exploit the physical and MAC layers. A jammer may be constant, deceptive, reactive, sweep-based, or directional; the effect can appear as packet loss, acknowledgement loss, increased delay, intermittent connectivity, or hidden terminal-like behaviour [6,7]. Detecting and localising the jammed area is itself non-trivial because observations are distributed and noisy [8,23]. For UAV missions, the attacker may also move, focus on a sector or target the command-and-control path rather than every link equally.

Anti-jamming methods include power control, directional antennas, coding, spread spectrum, frequency hopping, channel surfing, spatial retreat and cooperative routing around the affected area [14,15,24]. Cognitive radio broadens the design space by combining sensing, decision making and reconfiguration [11,12,13]. Machine-learning methods have also been widely studied for cognitive-radio control and adaptive policy selection [25,26,27,28]. More recent UAV and related mobile anti-jamming applications are discussed in Section 2.5 [29,30,31,32]. These approaches are promising, but their deployment depends on reliable observations and on the cost of reconfiguration. A policy that reacts too quickly may chase noise; one that reacts too slowly may lose mission-critical traffic before recovery begins.

This paper focuses on this policy layer. It does not claim to implement a complete cognitive-radio stack. Instead, it uses explicit, interpretable policies and measures their behaviour under repeatable mission scenarios. This approach provides a baseline for later reinforcement-learning or optimisation-based controllers.

### 2.3. Threat Model for Reconnaissance UAV Links

In reconnaissance missions, the radio link is not merely a transport service. It is part of the sensing chain because it carries sensor-tasking commands, position updates, health telemetry, relay coordination and selected payload products. The adversary does not need to destroy every packet to degrade mission value. It may be sufficient to delay a target handover, interrupt a return feed, break the relay path during a manoeuvre, or force the operators to reduce sensor tempo. These observations distinguish mission-network resilience from generic throughput maximisation.

Three threat properties are particularly relevant. The first is selectivity: a jammer may affect only one frequency, a narrow sector or a subset of links. The second is temporality: interference can be short, persistent or synchronised with a mission phase. The third is observability: a node may observe packet and acknowledgement loss without knowing whether the cause is distance, fading, congestion, environmental interference, or intentional action. Frequency manoeuvring is attractive because it can respond before a full network-layer reroute is required. Still, it is risky because a wrong decision can move the whole group into a new radio state.

The policy comparison in this paper is therefore framed as a mission-control problem rather than as a pure physical-layer problem. The tested policies represent common operational instincts: hold the assigned channel unless necessary, switch as soon as degradation is observed, wait for confirmation, hop proactively or reduce bandwidth to limit spectral overlap. The results should be interpreted as evidence of these operational instincts in a controlled scenario.

### 2.4. Network Experimentation and Emulation

Network simulation and emulation are complementary. Discrete-event simulators such as ns-3 provide controlled, scalable models, broad protocol support, and reproducibility [33,34]. Real-time emulators, in contrast, can execute actual applications and protocol stacks while modelling network effects. EMANE is designed as a modular wireless emulation framework with physical-layer and radio-model components for propagation, antenna and interference effects [35]. CORE provides a virtual-network environment for running real applications and protocols, including real-time scenarios and hardware-in-the-loop configurations [36].

This paper uses EMANE/CORE because the research question depends on the practical interactions among mobility, application traffic, link-quality assessment, and runtime radio-profile updates. The automated runner executes repeated scenarios and collects logs from communication events, connectivity snapshots and radio-environment assessments. This approach does not replace field trials, but it provides a controlled bridge between abstract anti-jamming models and operational software behaviour.

### 2.5. Positioning of This Paper

The reviewed literature provides strong foundations for UAV communication, cognitive radio, anti-jamming mechanisms and network experimentation. What is less common is a compact, repeatable comparison of simple frequency-control policies under mission-like UAV traffic with explicit switching cost and simultaneous environmental and adversarial interference. Many anti-jamming studies emphasise algorithmic optimality, while many UAV-network studies emphasise connectivity, routing or air-ground channel modelling. This work sits between those areas: it asks how much benefit a mission software layer can obtain from straightforward radio manoeuvring decisions before more complex learning, sensing or routing mechanisms are introduced.

Several recent studies extend this space they are compared in Table 1. Reinforcement-learning-based relay and power-allocation control for UAV swarms under jamming has been demonstrated in [29]; lightweight chaotic frequency-hopping sequence generation has been proposed to secure UAV links against jamming and spoofing [30]; multi-agent deep reinforcement learning has been applied to the anti-jamming spectrum access in satellite-linked settings with dynamics comparable to a mobile aerial relay [31]; and a 2025 survey formalises agent-based, perception–decision–action anti-jamming controllers for UAV communications [32]. Relative to [29,30,31,32], this paper does not propose a new learning or cryptographic mechanism; instead, it isolates and measures the operational pre-condition that any such controller must eventually satisfy—namely, when the expected recovery benefit of a manoeuvre exceeds its reconfiguration cost—using interpretable baseline policies under an explicit switching-cost model and a mission-level coverage-continuity metric that is largely absent from the cited work. On the experimentation side, EMANE/CORE was selected because it executes real application and protocol stacks against an emulated radio channel and supports real-time and hardware-in-the-loop configurations [35,36]. These capabilities are important when the quantity of interest is runtime policy-layer behaviour rather than an idealised protocol trace. Reference [37] provides a complementary comparison of the trade-offs between two discrete-event simulators, ns-3 and OMNeT++, but it does not directly compare either platform with EMANE/CORE.

The reviewed works show that UAV links are vulnerable to mobility, interference and jamming, and that cognitive or anti-jamming mechanisms can in principle react by changing radio parameters. However, they do not fully answer an operational question that appears before a sophisticated controller is deployed: when is it actually worth changing frequency or bandwidth, and when does the reconfiguration cost outweigh the expected recovery? This question is important for reconnaissance UAV networks because the mission value of a communication link depends not only on average throughput but also on whether command, telemetry and ISR messages remain timely and sufficiently continuous during degraded radio conditions.

This paper addresses this gap by building a controlled EMANE/CORE experiment in which the same UAV mission network is exposed to clean, environmental, jamming-only and combined-interference conditions. Instead of starting from an opaque optimisation or learning algorithm, this paper first evaluates interpretable baseline policies: staying on the assigned channel, switching immediately after quality degradation, delaying the switch until degradation persists, periodically hopping, and adapting bandwidth. This design makes it possible to separate the benefit of frequency manoeuvring from the cost of the manoeuvre itself. It also provides a practical reference point for later work, because any more advanced controller should improve on these transparent baselines under the same traffic, mobility and interference assumptions.

This positioning also explains the choice of metrics. PDR captures immediate message survival, latency, and jitter capture message timeliness, while coverage continuity captures whether the team remains a connected communication structure. These metrics map more directly to unmanned reconnaissance operations than raw throughput alone. The broader plan is to use the present results as a baseline for a mission-aware controller that will select channel, bandwidth and timing actions according to the detected disturbance type and mission phase. In that next step, reinforcement learning or optimisation can be introduced with clearer evidence of what it improves: not only packet delivery but also the operational trade-off between resilience, continuity and reconfiguration cost.

## 3. Materials and Methods

### 3.1. Experiment Plan

**Figure 1:**
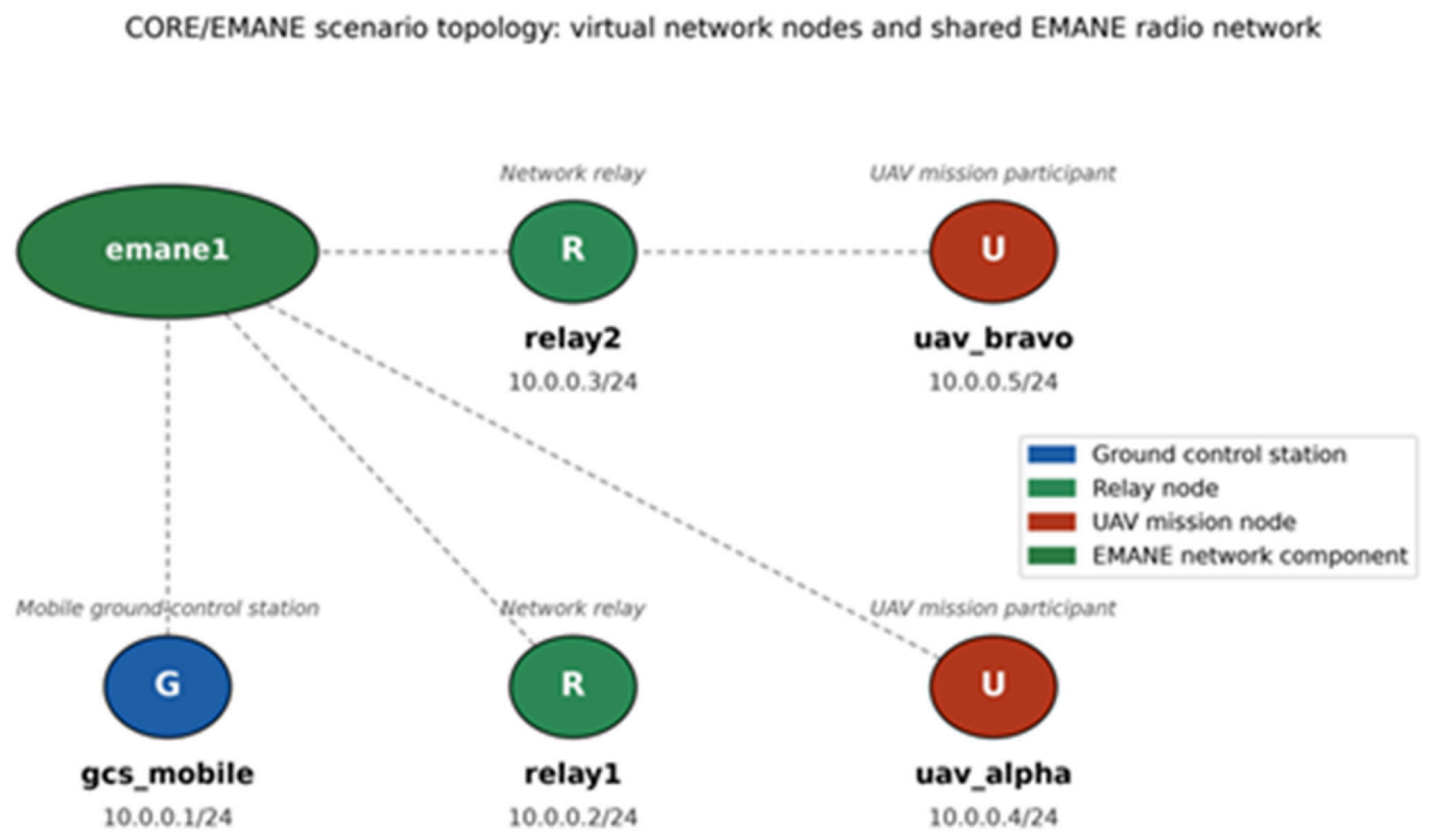
Schematic of the CORE/EMANE scenario topology, redrawn at full resolution from the original low-resolution CORE GUI capture for legibility. The diagram shows the initial placement of the ground control station, relay nodes, UAV nodes and the EMANE network component, and their IP addressing.

The experiment package contains 20 scenario variants generated for frequency-manoeuvring tests. Each scenario represents a simplified UAV mission network with five friendly participants(Figure 1): one mobile ground control station, two relay nodes and two UAV nodes. The ground control station acts as the command element, the relay nodes help maintain communication over distance, and the UAV nodes represent airborne mission assets. In CORE/EMANE, each participant is modelled as a separate virtual network node with its own position, IP address and radio profile.

The scenario is divided into 12 simulated time steps with each step representing 60 s of mission time. During every step, five communication events are generated between selected nodes, representing typical mission traffic such as commands, telemetry, relay messages and ISR updates. This gives 60 communication attempts in one run. To reduce randomness, each scenario is repeated 20 times, producing 400 successful runs and 24,000 attempted mission messages in total. Repetitions differ only in the pseudo-random seed governing mobility jitter, message timing and interference onset; the scenario-level metrics reported in Section 4 are therefore Monte Carlo estimates (empirical mean and standard deviation over 20 independent repetitions per scenario variant) rather than single-run outcomes.

In this paper, a node is a virtual network entity instantiated in the CORE/EMANE environment. Each node has a name, geographic position, operational status, IP address, EMANE interface, network-emulation module (NEM) identifier and radio profile (Table 2). A node may represent a UAV, a ground control station, a relay, or an interference source. The normal communication participants are gcs_mobile, relay1, relay2, uav_alpha and uav_bravo. Special nodes, such as weather_front and jammer_red, do not represent friendly mission participants; instead, they modify the radio environment by adding loss, delay, acknowledgement loss or frequency-selective disruption.

The scenario family contains four interference classes:

- **Clean**: no adversarial or environmental interference;
- **Environmental**: a non-adversarial disturbance such as a wideband weather or spectrum-occupancy front;
- **Jamming**: an intentional jammer with radius, strength, packet-loss, acknowledgement-loss, delay, frequency and bandwidth parameters;
- **Combined**: simultaneous environmental disturbance and intentional jamming.

**Figure 2:**
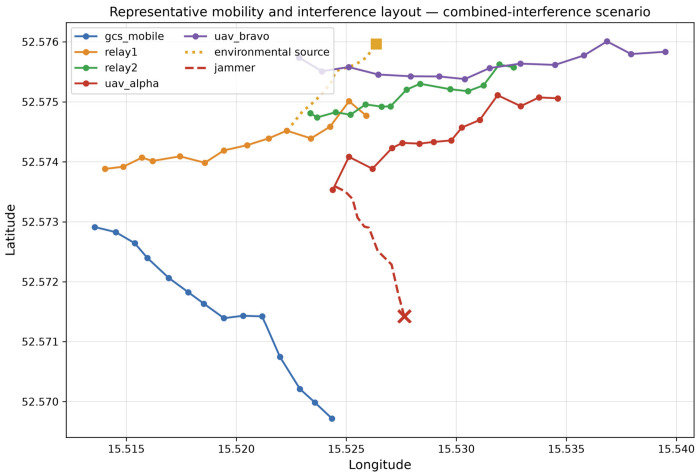
Representative mobility and interference layout for the combined-interference scenario redrawn at full resolution for legibility. Participant trajectories are shown with solid lines; jammer and environmental-source tracks are shown separately.

Figure 2 shows an example trajectory set from the combined-interference scenario. The scenario uses geographic coordinates near the mission area with altitudes ranging from 250 to 330 m. Participants exchange command, tasking, telemetry and ISR-update messages with critical or high mission priority. The coordinate window shown was selected to anchor the relative scenario geometry within a representative geographic extent and does not carry further operational significance.

### 3.2. Radio Profiles and Manoeuvring Policies

All policies start from 2437 MHz. The available channel set is 2412 MHz, 2437 MHz and 2462 MHz. These frequencies correspond to non-overlapping or weakly overlapping 2.4 GHz Wi-Fi-style channels under 20 MHz operation. There are several policy variants, which are outlined below:

- **Fixed-channel**: nodes do not change channel even when link quality degrades.
- **Immediate switch**: after one quality assessment below the threshold, the group selects the best available channel.
- **Delayed switch**: the group switches only after repeated poor connectivity observations.
- **Periodic hop**: the group changes channel every two scenario steps regardless of measured quality;
- **Adaptive bandwidth**: immediate switching is combined with bandwidth adaptation over 5 MHz, 10 MHz and 20 MHz.

The radio profile includes an explicit switching cost: an additional packet-loss probability of 0.12, an additional delay of 0.18 s and an additional jitter term of 0.04 s during reconfiguration. Table 3 summarises the policy parameters. These values constitute a deliberately simplified, radio-agnostic model of the transient penalty associated with channel reconfiguration. The assumed additional delay is broadly consistent with the timescale of channel switching and re-association in 802.11-class systems, which is considered in the context of refs. [21,22]. However, the complete parameter set was not calibrated against measurements from a particular radio platform and should therefore be interpreted as a controlled modelling assumption rather than a hardware-specific estimate. The values are held constant across the 5, 10 and 20 MHz bandwidth options to support a consistent comparison of the evaluated policies. Section 5.6 identifies hardware-based calibration and bandwidth-dependent switching costs as directions for future work.

### 3.3. Interference Model

The jammer profile includes an activation flag, radius, packet-loss contribution, acknowledgement-loss contribution, extra delay, strength, frequency, bandwidth and optional sector parameters. In the jamming scenarios, the jammer appears after the mission has already started, changes position and may retune its frequency. This approach produces a time-varying threat rather than a static blocked channel. In the present scenario pack, the sector parameter varied across the five jammer instances used in the jamming and combined-interference scenarios: three instances used a full 360° (omnidirectional) footprint and two instances used a 120° directional sector centred at 245°, so both the omnidirectional and the sectored jamming mode supported by the model were exercised in this campaign.

The environmental profile is modelled separately from adversarial jamming. It includes a radius, packet-loss contribution, acknowledgement-loss contribution, extra delay, strength, frequency, bandwidth and adjacent-channel leakage. In combined scenarios, the environmental source and the jammer coexist, creating overlapping and sometimes competing incentives for the frequency-control policy.

Table 4 reports the parameter ranges used to instantiate the environmental and jamming profiles across the twenty scenario variants.

### 3.4. Execution Workflow

Each scenario is executed from a clean CORE/EMANE session state. The control script sends simulation events by UDP, which are grouped by timestamp to avoid oversized datagrams. Node updates include geographic position, altitude, role, communication profile and optional jammer or environmental profile. Communication events are then injected as application-level UDP messages between named participants. The receiver-side listener and sender-side acknowledgement handling produce communication logs, while the radio-environment layer records link assessments and connectivity snapshots.

The batch runner repeats each scenario 20 times, and it aggregates results into an Excel workbook with four sheets: scenario descriptors, per-run repetition metrics, policy definitions, and generation configuration. This separation is important for reproducibility because scenario parameters and outcomes are kept in the same report. In the reported campaign, all 400 planned runs completed successfully.

### 3.5. Metrics

For each run, the runner collects message attempts, successfully delivered messages, dropped messages, link-quality assessments (Equations (1) and (2)), latency, jitter, connectivity snapshots and frequency manoeuvre events. The principal metrics are

(1) $$PDR=\frac{N_{delivered}}{N_{attempts}}$$

(2) Loss=1−PDR

Here, N_delivered_ denotes the number of messages successfully received by the intended recipient and N_attempts_ denotes the total number of message transmission attempts in the run. PDR is therefore the fraction of attempted messages that were successfully delivered, and Loss is its complement.

**Figure 3:**
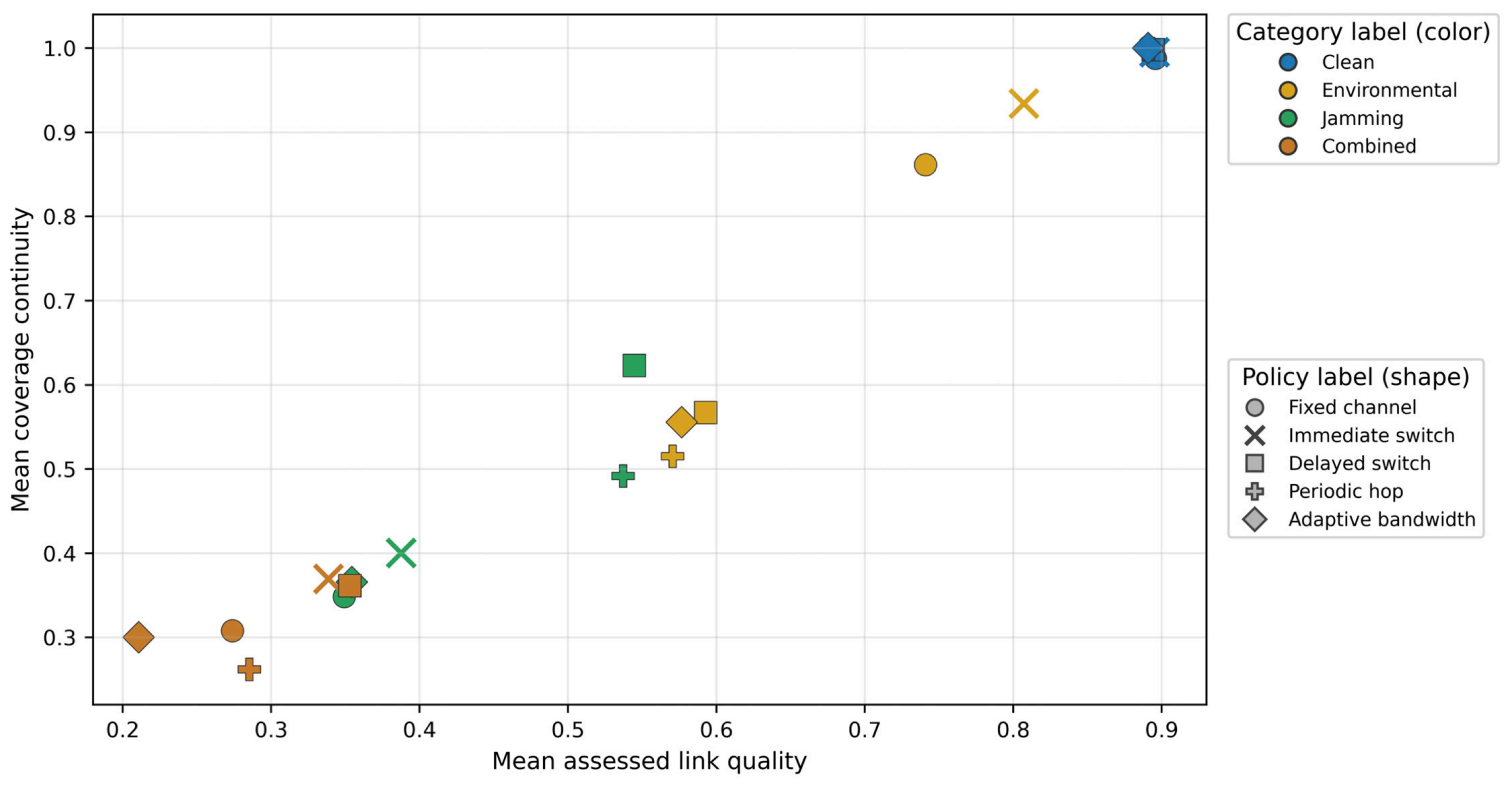
Relationship between mean assessed link quality and coverage continuity. Labels indicate policy families in non-clean scenarios.

Mean latency and jitter are derived from the radio assessment and communication logs. Coverage continuity is the fraction of participant-pair connectivity preserved in each snapshot, which is averaged over the run. The scenario-level values reported below are means across 20 repetitions; PDR uncertainty is shown using the empirical standard deviation across repetitions. A participant pair is counted as connected in a given snapshot when CORE/EMANE reports a viable radio link between the corresponding network-emulation module (NEM) interfaces, i.e., when the EMANE physical-layer model’s internal received-signal/link-viability check for the active waveform is satisfied; this determination is read directly from the EMANE PHY/MAC link state rather than recomputed independently from raw RSSI in post-processing. Coverage continuity is computed for each of the thirteen connectivity snapshots recorded per run (an initial snapshot plus one after each of the twelve simulated time steps) as the number of currently connected participant pairs divided by the ten possible unordered pairs among the five mission participants (gcs_mobile, relay1, relay2, uav_alpha, uav_bravo); the run-level value is the mean of this fraction across all snapshots in the run, and the scenario-level value reported in Table 5 and Table 6 is the mean across the 20 repetitions. The mean assessed link quality (Figure 3) is reported on the same 0–1 scale used for the switch-quality threshold (Table 3) and is averaged over all EMANE link-quality assessment events recorded during the run.

## 4. Results

### 4.1. Overall Packet Delivery

**Figure 4:**
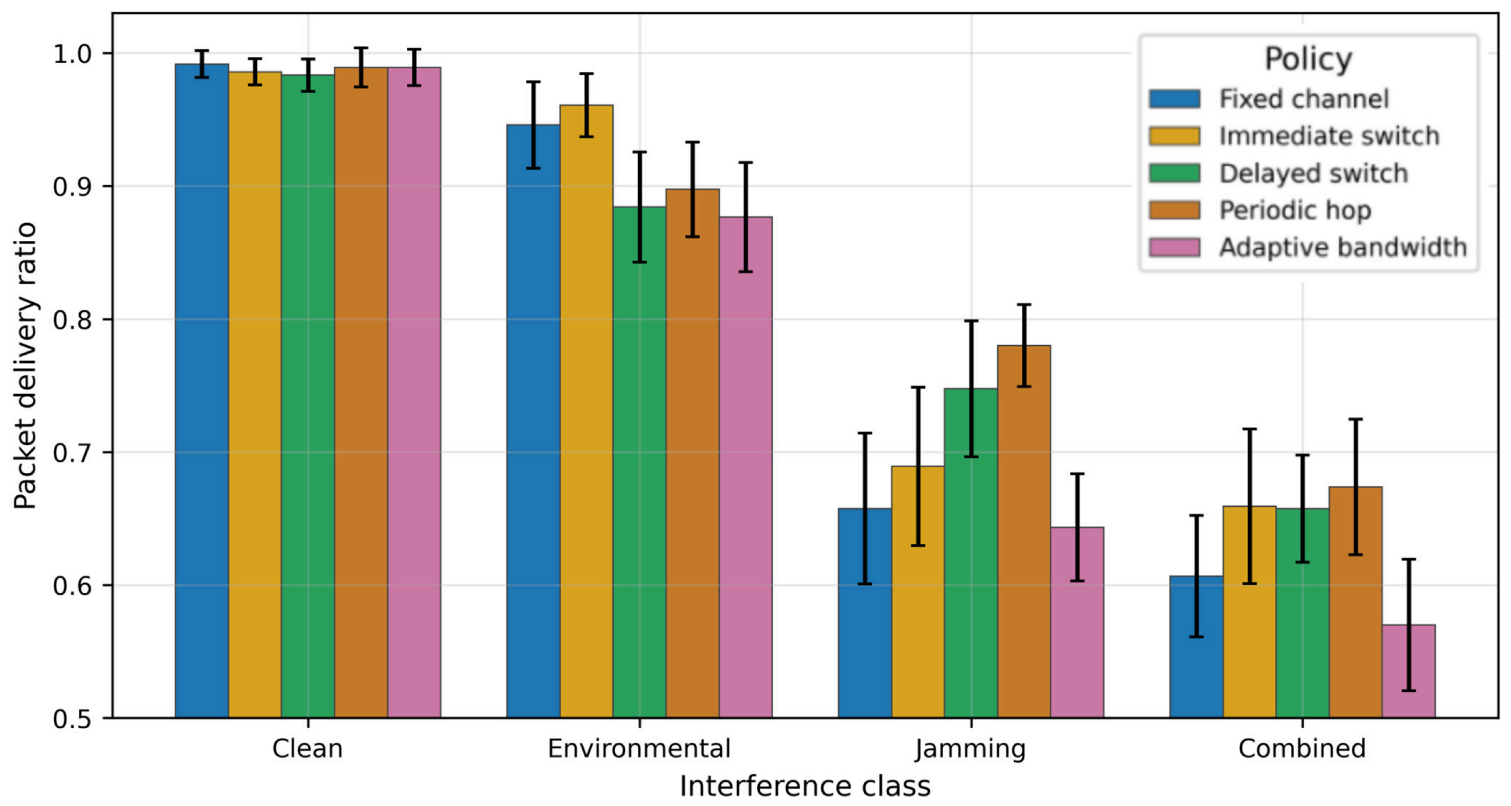
Packet delivery ratio by interference class and frequency manoeuvring policy. Error bars show standard deviation across 20 repetitions.

Figure 4 compares the PDR for all policies and interference classes. Clean conditions show that manoeuvring is unnecessary when the channel remains stable. Fixed-channel operation reached a PDR of 0.992. The other policies also performed well, but none improved on the fixed-channel baseline; this is expected because the switching logic can only add overhead when there is no persistent interference to avoid.

Under environmental interference, immediate switching achieved the best PDR of 0.961. This result represents a modest observed improvement over the fixed-channel value of 0.946 in the tested scenario. Delayed switching and adaptive bandwidth performed worse with PDR values of 0.884 and 0.877, respectively. Periodic hopping also underperformed immediate switching in this class, indicating that a targeted response better handled environmental disturbances than unconditional hopping.

Under jamming, the ranking changed. Periodic hopping achieved the highest PDR of 0.780, which was followed by delayed switching at 0.748 and immediate switching at 0.689. Fixed-channel operation reached only 0.658. The gain of periodic hopping over fixed-channel operation was therefore 12.2 percentage points. This result suggests that the jammer dynamics were sufficiently persistent and channel-focused for proactive channel movement to pay off despite switching costs.

The combined-interference class was the hardest. Periodic hopping again achieved the highest PDR, 0.674, but all policies were below 0.70. Immediate and delayed switching were close at 0.659 and 0.658. Adaptive bandwidth performed worst with a PDR of 0.570, indicating that bandwidth reduction alone does not guarantee resilience when multiple interference sources overlap in time and frequency.

### 4.2. Latency and Jitter

**Figure 5:**
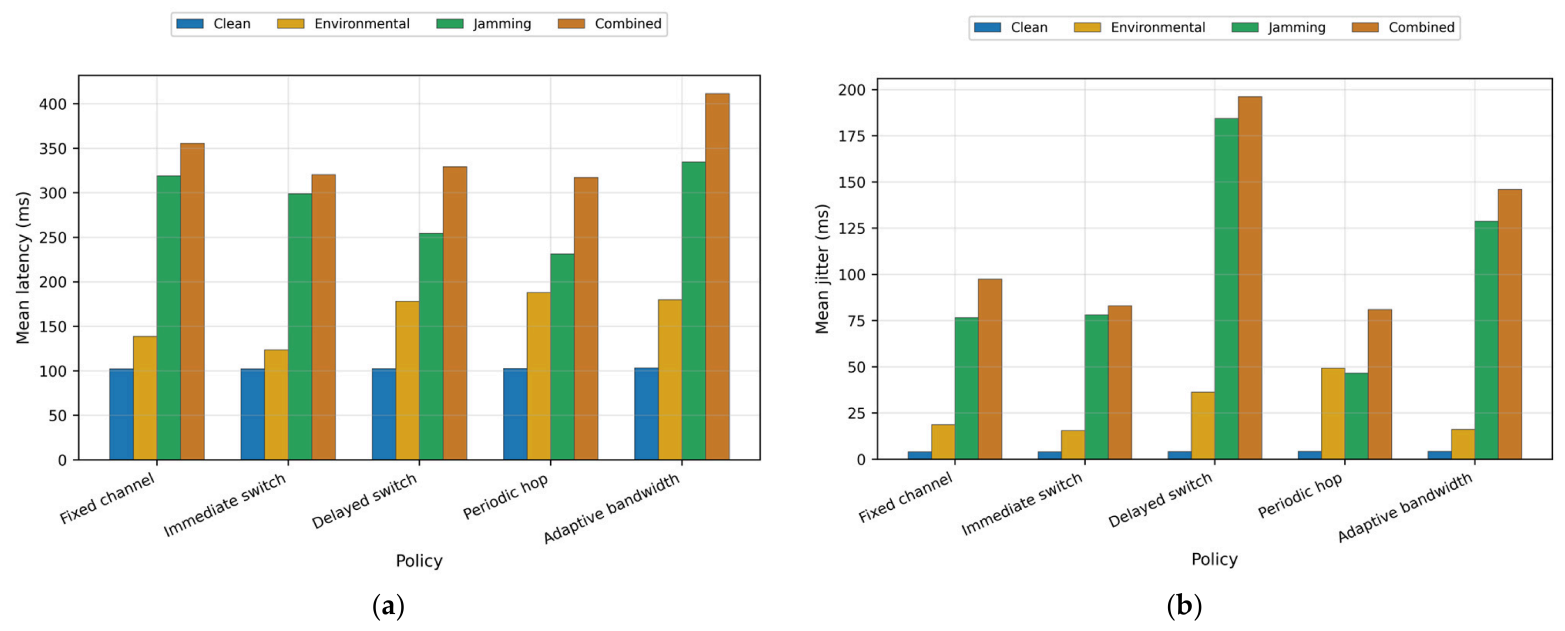
(**a**) Mean latency by policy and interference class; (**b**) Mean jitter by policy and interference class.

Figure 5 shows the effects of latency and jitter. Clean scenarios clustered near 102 ms latency and 4 ms jitter. Environmental interference increased both metrics, especially for delayed switching and periodic hopping. Immediate switching kept environmental latency at 123.6 ms compared with 138.7 ms for fixed-channel operation and 187.9 ms for periodic hopping.

In jamming-only scenarios, periodic hopping achieved the best PDR and also the lowest mean latency among the jamming cases: 231.4 ms. Delayed switching produced a competitive PDR, but its mean jitter was high at 184.3 ms. This observation reflects a recovery pattern in which the policy waits through repeated degraded observations before paying a reconfiguration cost. The policy, therefore, protects against premature switching but can accumulate timing variability during the degradation window.

Combined interference produced the largest delays. Adaptive bandwidth reached a mean latency of 411.4 ms and a jitter of 146.0 ms, explaining why its PDR result should not be interpreted as a mere throughput trade-off. In these scenarios, the bandwidth-adaptive policy appears to select conservative operating points too late or in the wrong interference geometry, resulting in both lower delivery and higher delay.

### 4.3. Coverage Continuity and Link Quality

**Figure 6:**
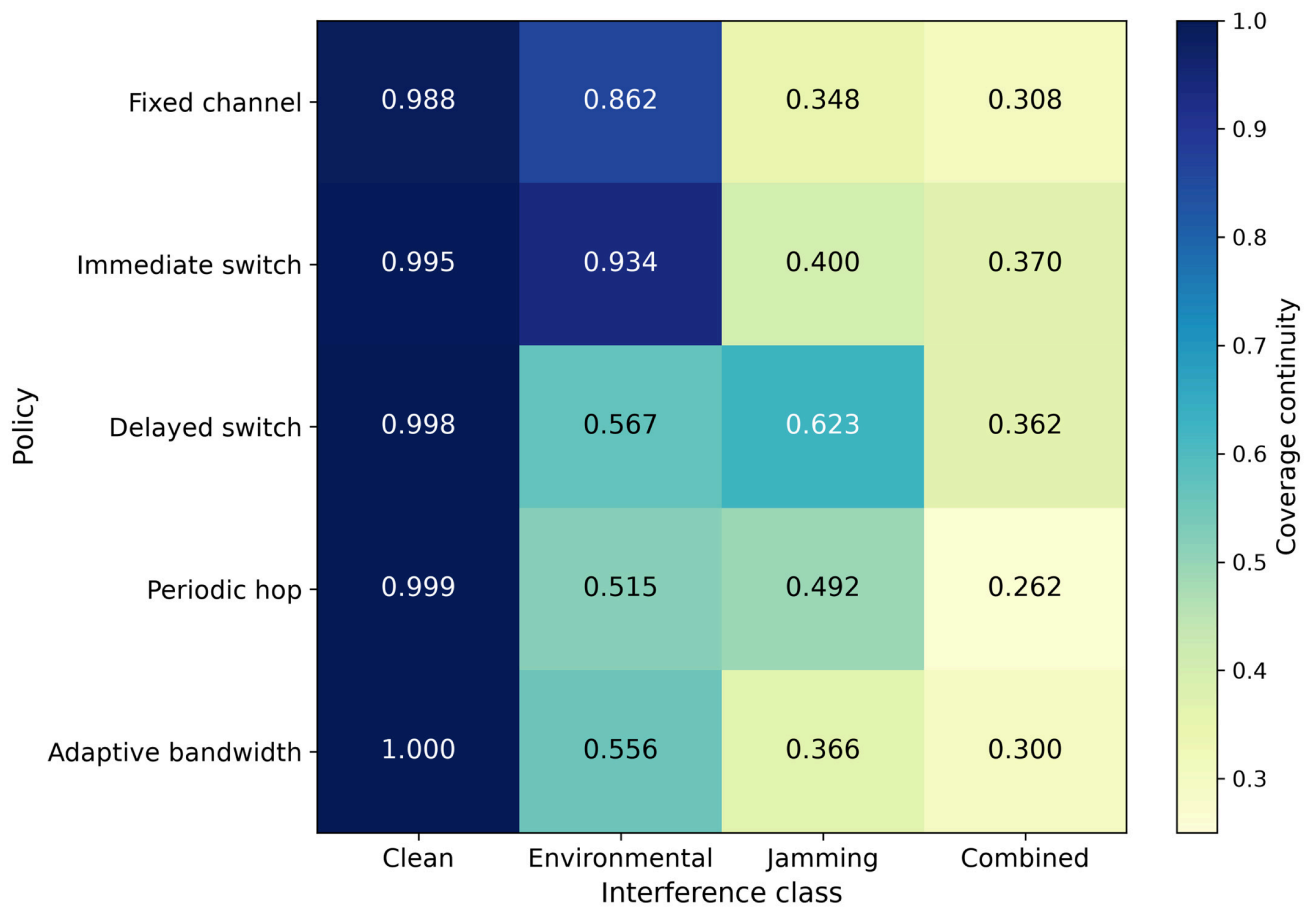
Coverage-continuity heatmap for the policy-interference matrix (companion to the PDR view in Figure 3). Higher values indicate better-preserved connectivity.

Coverage continuity provides a mission-level view that differs from PDR. Figure 3 plots the mean assessed link quality against mean coverage continuity. Clean scenarios remain near full coverage. Environmental interference disrupts policy separation: immediate switching maintained a mean coverage continuity of 0.934, whereas delayed switching and periodic hopping fell to 0.567 and 0.515, respectively. In jamming scenarios, delayed switching provided the highest coverage continuity (0.623), even though periodic hopping had the best PDR. This result indicates that delayed switching preserves more of the connectivity graph than periodic hopping with coverage-continuity values of 0.623 and 0.492, respectively, although periodic hopping delivers more messages with PDR values of 0.780 versus 0.748. Figure 6 presents the complete coverage-continuity matrix across all five policies and four interference classes, complementing the PDR view in Figure 3 and making explicit that the best-PDR policy in a class is not always the best-coverage policy.

Combined scenarios produced low coverage continuity for all policies. Immediate and delayed switching achieved values of 0.370 and 0.362, respectively. Fixed-channel operation and adaptive bandwidth achieved 0.308 and 0.300, while periodic hopping achieved 0.262. The best PDR policy in the combined class was therefore not the best coverage policy. This observation is operationally important: a mission planner may prefer a policy that preserves enough topology for command dissemination even if another policy wins on point-to-point packet delivery.

### 4.4. Policy and Scenario Summary

Table 5 summarises the best PDR policy in each interference class, while Table 6 reports the full matrix of scenario-level values. The results highlight that no single policy dominates across all operating conditions. The fixed channel is best when there is no interference; immediate switching is best for environmental disturbances; and periodic hopping is best under jamming and combined interference. The operational conditions for beneficial manoeuvring are therefore contextual rather than universal.

**Table 5:** Best PDR policy by interference class.

Interference Class	Policy	PDR	Latency (ms)	Jitter (ms)	Coverage
Clean	Fixed-channel	0.992	102.3	4.1	0.988
Environmental	Immediate switch	0.961	123.6	15.5	0.934
Jamming	Periodic hop	0.780	231.4	46.6	0.492
Combined	Periodic hop	0.674	317.2	81.0	0.262

Table 5 reports point estimates for the single best-performing PDR policy in each interference class, whereas Table 6 reports the complete matrix of mean values for all five policies across all four interference classes. PDR is additionally reported as the mean ± standard deviation over the 20 repetitions per variant. The values in Table 5 are a numerically consistent subset of those in Table 6.

The drop counts in Table 6 correspond to 1200 attempts per scenario variant. In clean scenarios, drops range from 10 to 20; in combined scenarios, they range from 372 to 516. The magnitude of this shift confirms that the scenario pack meaningfully stresses the communication system.

**Table 6:** Scenario-level summary over 20 repetitions per variant.

Interference Class	Policy	PDR	Latency (ms)	Jitter (ms)	Coverage	Drops
Clean	Fixed-channel	0.992 ± 0.010	102.3	4.1	0.988	10
Clean	Immediate switch	0.986 ± 0.010	102.3	4.1	0.995	17
Clean	Delayed switch	0.983 ± 0.012	102.4	4.2	0.998	20
Clean	Periodic hop	0.989 ± 0.015	102.6	4.2	0.999	13
Clean	Adaptive bandwidth	0.989 ± 0.014	103.3	4.3	1.000	13
Environmental	Fixed-channel	0.946 ± 0.032	138.7	18.7	0.862	65
Environmental	Immediate switch	0.961 ± 0.024	123.6	15.5	0.934	47
Environmental	Delayed switch	0.884 ± 0.041	178.2	36.3	0.567	139
Environmental	Periodic hop	0.898 ± 0.036	187.9	49.3	0.515	123
Environmental	Adaptive bandwidth	0.877 ± 0.041	179.9	16.1	0.556	148
Jamming	Fixed-channel	0.658 ± 0.057	319.1	76.6	0.348	411
Jamming	Immediate switch	0.689 ± 0.060	299.0	78.1	0.400	373
Jamming	Delayed switch	0.748 ± 0.051	254.5	184.3	0.623	303
Jamming	Periodic hop	0.780 ± 0.031	231.4	46.6	0.492	264
Jamming	Adaptive bandwidth	0.643 ± 0.040	334.6	128.7	0.366	428
Combined	Fixed-channel	0.607 ± 0.046	355.4	97.5	0.308	472
Combined	Immediate switch	0.659 ± 0.058	320.4	83.0	0.370	409
Combined	Delayed switch	0.658 ± 0.040	329.2	196.2	0.362	411
Combined	Periodic hop	0.674 ± 0.051	317.2	81.0	0.262	372
Combined	Adaptive bandwidth	0.570 ± 0.049	411.4	146.0	0.300	516

Note: Values in the PDR column are reported as mean ± standard deviation over 20 repetitions for each scenario and policy variant. The term after ± indicates the variability of the obtained results across repetitions.

## 5. Discussion

### 5.1. Operational Conditions for Beneficial Manoeuvring

The clean scenarios establish the lower bound for intervention: if link quality is high and stable, channel changes are unnecessary. Even small switching costs can make an adaptive policy inferior to a fixed channel. Therefore, manoeuvring should be treated as an operational response to observed or predicted degradation rather than as a default good.

The environmental scenarios show that immediate switching is useful when the disturbance is sufficiently localised in both frequency and time and when the quality estimate is reliable. The PDR improvement over fixed-channel operation is not dramatic, but latency and coverage also improve. This combination is important because it means immediate switching does not merely rescue a few packets; it improves the overall mission communication state.

The jamming scenarios show a different set of operating conditions under which manoeuvring becomes beneficial. Periodic hopping performs best because the jammer is persistent enough that waiting for detection can result in packet loss. Delayed switching remains valuable for coverage continuity, suggesting it helps avoid unnecessary group moves. These results imply that a practical controller should be able to classify the disturbance type. A reactive policy suitable for environmental interference may be too conservative against an intentional jammer.

### 5.2. Adaptive Bandwidth Result

The adaptive-bandwidth policy was expected to trade capacity for robustness. In the tested scenarios, it did not achieve this goal. The policy performed acceptably in clean conditions but degraded under environmental, jamming and combined interference. This does not invalidate adaptive bandwidth in general; rather, it shows that the simple tested rule is insufficient. Narrowing the bandwidth can reduce overlap with interference. Still, it can also reduce throughput, increase the relative impact of reconfiguration, and cause failures when the disturbance remains aligned with the selected channel.

One plausible explanation is that narrowing the channel may increase the airtime required to carry the same message payload, thereby lengthening each transmission’s exposure window to an ongoing disturbance. Combined with the fixed reconfiguration cost paid on every bandwidth change, this may cause the policy to incur repeated switching overhead without a compensating reduction in frequency-domain overlap.

Future work should treat bandwidth as a joint optimisation variable with channel, route, traffic priority and mission state. For example, a controller could select a narrow bandwidth only for critical low-rate telemetry during high-risk intervals while restoring wider channels for bulk ISR payloads when the interference estimate improves.

### 5.3. Coverage Versus Delivery

The difference between PDR and coverage continuity is one of the main findings. Periodic hopping wins under jamming and combined interference by PDR but not always by coverage. A reconnaissance mission may be concerned with both. High PDR on a subset of links may be sufficient for telemetry relay, but poor coverage can isolate assets, reduce redundancy and complicate command dissemination. Conversely, preserving the connectivity graph with delayed switching may be useful even if individual messages experience higher jitter.

These insights suggest that anti-jamming policies should not be selected using a single scalar metric. A mission-aware objective could weight PDR, latency, jitter, and coverage differently depending on the phase: launch, ingress, sensor collection, target handover, return, or emergency recovery.

### 5.4. Relevance to Reconnaissance and Jamming Challenges

The special-issue theme emphasises reconnaissance, jamming and unmanned-vehicle capabilities. The presented experiment directly links these domains. UAV reconnaissance depends on a persistent information flow; jamming attacks target that flow. Frequency manoeuvring is a controllable capability that can be implemented in mission software. The results indicate that the capability is valuable but only when matched to the interference mode. Blind adaptation is not enough.

### 5.5. Future Research Directions

The next step is to replace the static policy choice with a mission-aware controller. Such a controller should estimate whether degradation is random, environmental, or adversarial and then select a channel, bandwidth, and timing action according to the mission phase. Reinforcement learning is a candidate method, but a learned controller should be trained against interpretable baselines such as the policies evaluated here. Otherwise, it is difficult to know whether the learned policy improves because it has discovered a useful electromagnetic response or because it exploits a narrow artefact of the scenario generator.

Another direction is multi-layer manoeuvring. Frequency change can be coordinated with relay selection, routing, transmit-power control, antenna pointing and traffic prioritisation. For example, a UAV carrying high-value imagery may remain on a robust narrow channel for control traffic while delaying bulk payload transfer until the relay geometry improves. Similarly, the network may accept lower coverage continuity during a short ingress phase if PDR for command links remains high, but it prefers coverage preservation during distributed search.

Finally, the interference model should be calibrated against measured spectra and packet traces. EMANE/CORE provides a useful experimental substrate, but the credibility of operational conclusions depends on realistic loss, delay, and retuning behaviour. Field trials with software-defined radios and controlled jamming or benign interference sources would allow the switching-cost and channel-overlap assumptions to be refined.

### 5.6. Limitations

This paper has several limitations. First, the scenarios use a controlled five-participant topology. Larger swarms may exhibit different contention, routing, and coordination behaviour. Second, the switching-cost model is explicit but simplified. Real radios may incur hardware-dependent retuning time, synchronisation loss, regulatory constraints and waveform-specific overhead. Third, the jammer and environmental profiles are parameterised models rather than measured radio-frequency recordings. Fourth, the evaluation focuses on communication metrics rather than directly modelling mission success. Finally, the current policies are interpretable rules—not learned or optimised controllers. Routing coupling is intentionally out of scope: as noted in Section 2.1, this paper isolates the frequency/bandwidth policy layer from routing so that measured effects are not confounded by route reconvergence; evaluating manoeuvring jointly with a dynamic routing protocol is identified as future work. The EMANE/CORE workflow itself is not limited to five nodes and can be extended to larger swarms without methodological changes, but the present campaign does not provide evidence about contention or coordination behaviour at that scale. Finally, the switching-cost model used here does not vary with bandwidth (Section 3.2); a bandwidth-dependent cost model is a direct extension once hardware-calibrated retuning data are available.

These limitations define a useful next stage. The same EMANE/CORE workflow can be extended with more nodes, heterogeneous radio models, directional antennas, route adaptation, realistic spectrum traces and reinforcement-learning policy selection. Field measurements should be used to calibrate the packet-loss, acknowledgement-loss and delay components of the interference model.

## 6. Conclusions

This paper evaluated five frequency-manoeuvring policies for UAV network communication under clean, environmental, jamming-only, and combined-interference conditions. The EMANE/CORE-based workflow executed 20 scenario variants with 20 repetitions each and measured PDR, latency, jitter, packet loss, link quality and coverage continuity.

The results show that manoeuvring is condition-dependent. Fixed-channel operation is best in clean conditions, immediate switching is best under environmental interference, and periodic hopping is best under jamming and combined interference. Delayed switching provides a useful middle ground for preserving coverage continuity, especially under jamming. Adaptive bandwidth, as implemented here, did not improve resilience under contested conditions and requires a more mission-aware selection rule. Future work should calibrate the switching-cost and interference models against field measurements and extend the comparison to a mission-aware, learning-based controller trained against the interpretable baselines established here (Section 5.5).

The operational implication is clear: a UAV network should not use a single static anti-jamming policy for all phases and threat types. Instead, the controller should identify whether degradation is transient, environmental, adversarial or combined and then select a manoeuvring policy whose switching cost is justified by the expected recovery benefit.

Wearable cardioverter defibrillators implemented early after the onset of cardiac disease offer valuable physiologic and behavioural insights through the integration of TRENDS RMS data with WCDs, and they may facilitate earlier interventions for HF and arrhythmia risk, highlighting the potential role of the use of these devices as both life-saving devices and comprehensive remote monitoring tools.

## Figures and Tables

**Table 1:** Comparison of representative recent anti-jamming studies in UAV and related mobile aerial/space networks (2023–2025) with this paper.

Study	Year	Platform/Method	Interference Modelled	Switching Cost Modelled	Metric(s)	Key Differentiator
Lv et al. [29]	2023	Multi-agent RL (simulation)	Jamming	No	Throughput/reward	Learned relay and power allocation
Atheeq et al. [30]	2024	Chaotic frequency hopping (analytical/sim.)	Jamming, spoofing	No	BER/detection resistance	Cryptographically unpredictable hop sequence
Cao et al. [31]	2025	Multi-agent DRL (LEO satellite sim.)	Jamming	No	Spectrum-access success	Learned spectrum access in a mobile aerial/space relay
Yang et al. [32]	2025	Survey/taxonomy	Jamming (agent-based)	Partially (qualitative)	N/A (survey)	Perception–decision–action controller taxonomy
This paper	2026	EMANE/CORE emulation	Clean, environmental, jamming, combined	Yes (explicit loss/delay/jitter)	PDR, latency, jitter, coverage continuity	Interpretable baseline manoeuvring policies with explicit switching cost and mission-level continuity

**Table 2:** Node roles used in the CORE/EMANE scenario model.

Node Name	Role	Operational Meaning
gcs_mobile	Mobile ground-control station	Command centre that sends tasking and receives reports
relay1, relay2	Network relays	Intermediate nodes that preserve GCS–UAV connectivity
uav_alpha, uav_bravo	UAV mission participants	Airborne nodes sending telemetry, ISR updates and alerts
weather_front	Environmental source	Non-adversarial disturbance affecting loss, delay and ACK quality
jammer_red	Intentional jammer	Adversarial node with radius, strength, sector and frequency profile

**Table 3:** Frequency manoeuvring policy parameters used in the scenario pack.

Policy	Freq. Set (MHz)	BW Set (MHz)	$q_{sw}$ ^1^	$d_{sw}$ ^2^	Hop Step
Fixed-channel	2437	20	0.45	2	-
Immediate switch	2412, 2437, 2462	10, 20	0.58	1	-
Delayed switch	2412, 2437, 2462	10, 20	0.52	2	-
Periodic hop	2412, 2437, 2462	20	0.45	1	2
Adaptive bandwidth	2412, 2437, 2462	5, 10, 20	0.60	1	-

^1^ $q_{sw}$ denotes the link-quality threshold below which a policy is allowed to trigger a frequency or bandwidth manoeuvre. ^2^ $d_{sw}$ denotes the number of consecutive degraded quality assessments required before the manoeuvre is executed.

**Table 4:** Interference-profile parameters used in the experimental campaign.

Parameter	Environmental	Jamming	Combined
Onset time (s)	180	240	180 (environmental)/240 (jamming)
Radius (m)	1500	950	as environmental/as jamming
Packet-loss contribution	0.24–0.32	0.72–0.88	as environmental/as jamming
ACK-loss contribution	0.18–0.22	0.78–0.90	as environmental/as jamming
Additional delay (s)	0.18–0.30	0.42–0.58	as environmental/as jamming
Strength (normalised)	0.55–0.87	1.00–1.48	as environmental/as jamming
Centre frequency (MHz)	2437–2452	2412–2437	as environmental/as jamming
Bandwidth (MHz)	12–35	8–40	as environmental/as jamming
Sector	N/A (omnidirectional source)	360 degrees (3 of 5 instances) or 120 degrees centred at 245 degrees (2 of 5 instances)	as jamming

Note: values denote the minimum–maximum range used across the five parameterised scenario instances generated for each interference class (one instance per manoeuvring-policy variant); combined-interference scenarios apply the environmental and jamming values of the matching instance index simultaneously. Two of the five jamming/combined instances used a 120-degree directional sector centred at 245 degrees; the remaining three used a full 360-degree omnidirectional footprint. Adjacent-channel leakage is not separately logged in the scenario-generation configuration and is therefore omitted from this table. Onset time and radius are constant across instances within each source type.

## Data Availability

Data are not publicly available due to the classified nature of the work. Aggregated, non-attributable scenario-level results (Table 5 and Table 6) are provided within the manuscript to support independent verification of the reported trends.
